# Latent class evaluation of three serological tests for the diagnosis of human brucellosis in Bangladesh

**DOI:** 10.1186/s41182-016-0031-8

**Published:** 2016-10-04

**Authors:** A. K. M. A. Rahman, C. Saegerman, D. Berkvens

**Affiliations:** 1 Department of Medicine, Bangladesh Agricultural University, Mymensingh, 2200 Bangladesh; 2 Research Unit of Epidemiology and Risk Analysis applied to the Veterinary Sciences (UREAR-ULg), Department of Infectious and Parasitic Diseases, Faculty of Veterinary Medicine, University of Liège, Liège, Belgium; 3 Department of Biomedical Sciences, Institute of Tropical Medicine, Antwerpen, Belgium

## Abstract

**Background:**

A Bayesian latent class evaluation was used to estimate the true prevalence of brucellosis in livestock farmers and patients with prolonged pyrexia (PP) and to validate three conditionally dependent serological tests: indirect ELISA (iELISA), Rose Bengal Test (RBT), and standard tube agglutination (STAT). A total of 335 sera from livestock farmers and 300 sera from PP patients were investigated.

**Results:**

The true prevalence of brucellosis in livestock farmers and PP patients was estimated to be 1.1 % (95 % credibility interval (CrI) 0.1–2.8) and 1.7 % (95 % CrI 0.2–4.1), respectively. Specificities of all tests investigated were higher than 97.8 % (95 % CrI 96.1–99.9). The sensitivities varied from 68.1 % (95 % CrI 54.5–80.7) to 80.6 % (95 % CrI 63.6–93.8). The negative predictive value of all the three tests in both populations was very high and more than 99.5 % (95 % CrI 98.6–99.9). The positive predictive value (PPV) of all three tests varied from 27.9 % (95 % CrI 3.6–62.0) to 36.3 % (95 % CrI 5.6–70.5) in livestock farmers and 39.8 % (95 % CrI 6.0–75.2) to 42.7 % (95 % CrI 6.4–83.2) in patients with PP. The highest PPV were 36.3 % for iELISA and 42.7 % for RBT in livestock farmers and pyrexic patients, respectively.

**Conclusions:**

In such a low prevalence scenario, serology alone does not help in diagnosis and thereby therapeutic decision-making. Applying a second test with high specificity and/or testing patients having history of exposure with known risk factors and/or testing patients having some clinical signs and symptoms of brucellosis may increase the positive predictive value of the serologic tests.

**Electronic supplementary material:**

The online version of this article (doi:10.1186/s41182-016-0031-8) contains supplementary material, which is available to authorized users.

## Background

Brucellosis is a bacterial zoonosis affecting both human and animal health [1]. It is an occupational hazard for livestock farmers, milkmen, butchers, hired animal caretakers, and veterinarians [2]. Fever, sweating, fatigue, headache, and joint pain are important non-specific symptoms of brucellosis in humans. Brucellosis in humans is often misdiagnosed due to its unspecific clinical symptoms similar to that of other endemic pyrexic diseases like tuberculosis, malaria, typhoid, or rheumatic fever. Several sero-prevalence studies from Bangladesh indicate that the apparent prevalence of brucellosis in risk groups varies from 4.4 to 12.8 % [3–5]. The Rose Bengal Test (RBT), standard tube agglutination (STAT), and ELISA either alone or in combination were used for those studies. None of these tests is perfect, and thus, they cannot be used to estimate true prevalences. In the absence of a reasonable gold standard test, simultaneous estimation of true prevalence and test validation can be performed by applying multiple diagnostic tests to every individual using a Bayesian latent class analysis framework allowing the combination of test results and external information [6–8]. While evaluating the results of multiple diagnostic tests, it is essential to consider whether or not the tests can be assumed conditionally independent of each other given the true disease status. Assuming conditional independence may lead to biased estimates for test characteristics if the tests are conditionally dependent [9, 10]. As indirect ELISA (iELISA), RBT, and STAT are based on the same biological phenomenon [11], i.e., detection of anti-Brucella smooth lipopolysaccharide (sLPS) antibodies, they can primarily be considered as conditionally dependent [12]. Up to our knowledge, a latent class analysis was not used yet for the evaluation of multiple serological tests to diagnose human brucellosis.

The aims of this study were to estimate the true prevalence for brucellosis in two study groups and to evaluate three conditionally dependent serological tests using latent class analysis.

## Methods

### Study population, study area, and sampling strategy

Blood samples of livestock farmers were collected between September 2007 and August 2008 in Mymensingh district. Three hundred and thirty-five livestock owners or hired animal caretakers agreed to participate. The details of the livestock farmers included in this study were described in a previous paper [13]. In brief, out of a total of the 146 unions (sub Upa-Zilla) of Mymensingh district, 28 were randomly selected. One geographical coordinate was randomly selected from each selected union and located by a hand-held GPS reader. Livestock farmers within 0.5 km radius of the selected point were informed about the survey, and those who agreed were sampled.

Blood samples from prolonged pyrexia (PP) patients were taken randomly once in a week at Mymensingh Medical College (MMC) hospital. These patients originated from Mymensingh and neighboring districts like Netrakona, Jamalpur, Sherpur, and Tangail. Patients with PP were defined having a body temperature higher than 38 °C for a 3-week period. Every day, approximately 100 patients visit the outdoor facility of the hospital. Patients who met the inclusion criterion were asked for a blood sample. In addition, hospitalized patients meeting the inclusion criterion at the same day were also asked for a sample. A total of 300 PP blood samples were collected from October 2007 to May 2008.

### Collection and handling of blood samples

The collection and handling of blood samples was described in a previous paper by Rahman et al. [13]. In brief, about 4 mL of blood was collected with disposable needles and Venoject tubes, labeled, and transported to the laboratory on ice (after clotting) within 12 h of collection. The blood samples were kept in refrigerator (2–8 °C), and 1 day later, sera were separated by centrifuging at 6000*g* for 10 min.

### Serological tests

All blood samples were tested in parallel by indirect IgG iELISA, RBT, and STAT at the Medicine Department laboratory of Bangladesh Agricultural University, Mymensingh, Bangladesh. RBT was performed as described by Alton et al. [14]. The STAT was carried out on doubling dilutions of serum from 1:20 to 1:320 according to Alton et al. [14]. *Brucella abortus* and *Brucella melitensis* antigens (Cypress Diagnostics, Langdorpsesteenweg 160, B-3201, Belgium) were used according to the instruction of the manufacturer. Titres ≥1:160 were considered as positive. The iELISA was used as described by Limet et al. [15] using *B. abortus* biotype 1 antigen (Strain Weybridge 99, A epitope). Six dilutions of positive control serum no. 1121 (1/270–1/8340, corresponding to 2–60 units) were used to generate a standard curve. The detail procedure was described in a previous paper by Rahman et al. [13].

### Statistical analysis

In order to determine the true prevalence, sensitivity, and specificity of the three tests for two subpopulations, Bayesian latent class analysis was performed using a multinomial model, based on conditional probabilities [16]. The full model assuming conditional dependence is overparameterized. It thus requires external (prior) information for prevalence and test characteristics (sensitivity and specificity). Prior information on prevalence [3–5] and sensitivity and specificity of iELISA [17] was extracted from published reports, and three other conditional probabilities adapted by experts of the Department of Infectious and Parasitic Diseases, Faculty of Veterinary Medicine, University of Liège, Belgium (Table 1) were included. The beta prior distribution was considered in Bayesian model. The analysis was conducted in WinBUGS 1.4 [18] and R 3.2.2 (R Foundation and Statistical Computing 2015). The model was run with a burn-in of 50,000 iterations, and estimates were based on a further 50,000 iterations and three chains. The posterior predictive *P* value, the Deviance Information Criterion (DIC), and the number of parameters effectively estimated by the model (pD)) were used to assess the fit between the prior information and the test results [16]. The WinBUGS code for conditional dependence of three tests for a two populations Bayesian model is shown in Additional file 1: Appendix A.

**Table 1:** Prior information for the Bayesian latent class evaluation of three serological tests for the diagnosis of brucellosis in livestock farmers and prolonged pyrexia patients (Beta distribution)

Conditional probabilities	Prior information (alpha, beta)
Prevalence (pr[1] and pr[2] in the model in Additional file 1: Appendix A) [13–15]	(1.57, 29.19)
Sensitivity of the iELISA for the diagnosis of sero-positive individuals (th1[2] and th2[2] in the model in Additional file 1: Appendix A[29]	(32.53, 14.51)
Specificity of the iELISA for the diagnosis of sero-negative individuals (th1[3] and th2[3] in the model in Additional file 1: Appendix A) [29]	(294.08, 6.98)
Probability to have a positive result for the RBT if the individual is sero-positive and positive for the iELISA (th1[4] and th2[4] in the model in Additional file 1: Appendix A)	(143.50, 10.09)
Probability to have a positive result for the STAT if the individual is sero-positive and positive for the iELISA and RBT (th1[8] and th2[8] in the model in Additional file 1: Appendix A)	(313.97, 10.68)
Probability to have a negative result for the STAT if the individual is sero-negative and negative for the iELISA and RBT (th1[12] and th2[12] in the model in Additional file 1: Appendix A)	(999.99, 6.02)

*iELISA* indirect ELISA, *RBT* Rose Bengal test, *STAT* standard tube agglutination test

The positive predictive value (PPV) and negative predictive value (NPV) of the tests were calculated using Eqs. 1 and 2 in Bayesian model.

1 $$ \mathrm{P}\mathrm{P}\mathrm{V}=\frac{\mathrm{Se}\mathrm{nsitivity}\ \left(\mathrm{S}\mathrm{e}\right)\ast \mathrm{P}\mathrm{revalence}\ \left( \Pr \right)}{\mathrm{Se}\ast \Pr +\left(1-\mathrm{Specificity}\ \left(\mathrm{S}\mathrm{p}\right)\right)*\left(1- \Pr \right)} $$

2 $$ \mathrm{N}\mathrm{P}\mathrm{V}=\frac{\mathrm{Sp}\ast \left(1- \Pr \right)}{\left(1-\mathrm{S}\mathrm{e}\right)\ast \Pr +\mathrm{S}\mathrm{p}\kern0.1em \left(1- \Pr \right)} $$

### Sensitivity analyses

The influence of the prior information on the estimates of the characteristics of the diagnostic tests was verified using sensitivity analysis. This was done by using uniform priors and slight perturbations (in steps of 10 or 15 %) of the prior intervals [18].

## Results

### Descriptive statistics

Cross-classified results of the three serological tests are shown in Table 2. From the 335 livestock farmers, only 0.6 % were positive and 97.3 % were negative in all three tests. The apparent prevalence of brucellosis among livestock farmers based on a parallel interpretation of the three tests was 2.7 %. Of 300 PP patients, only 2.0 % were positive and 97.3 % were negative in all three tests. Based on a parallel interpretation (if positive in at least one test), the apparent prevalence of brucellosis among PP patients was 2.7 %; 32.8 % (110/335) of the livestock farmers and 33.7 % (101/300) of the PP patients were female. All livestock farmers had contact with cattle (66.0 %) or goats (17.3 %) or with both (16.7 %). Among PP patients, only 27 % (81/300) had contact with cattle and or goats.

**Table 2:** Cross-classified test results of three serological tests applied on livestock farmers and prolonged pyrexia patients in Bangladesh

iELISA	RBT	STAT	Livestock farmers	PP patients
1	1	1	2	6
1	1	0	0	0
1	0	1	0	0
1	0	0	0	0
0	1	1	3	1
0	1	0	1	0
0	0	1	3	1
0	0	0	326	292
Total			335	300

*1* positive test result, *0* negative test result, *iELISA* indirect enzyme-linked immunosorbent assay, *RBT* Rose Bengal Test, *STAT* standard tube agglutination test, *PP* prolonged pyrexia

### Posterior estimates

The true prevalence of brucellosis among livestock farmers and PP patients is presented in Table 3. The true prevalence of brucellosis in livestock farmers and PP patients estimated were 1.1 % (95 % (CrI 0.1–2.8) and 1.7 % (95 % CrI 0.2–4.1), respectively. The performance of all three tests was similar in both populations. In both groups, specificity of all tests was greater than 97.8 % (95 % CrI 96.2–99.9). The sensitivity of iELISA, RBT, and STAT varied from 68.1 % (95 % CrI 54.5–80.7) to 69.6 % (95 % Cr1 56.0–81.6), 79.4 % (95 % CrI 59.5–95.0) to 79.2 % (95 % CrI 60.3–94.8), and 80.5 (95 % CrI 63.1–93.8) to 80.6 (95 % CrI 63.6–93.8) in livestock farmers and PP patients, respectively.

**Table 3:** Estimates of true prevalence, sensitivity, specificity of three serological tests used for the diagnosis of brucellosis in livestock farmers and PP patients in Bangladesh

		Livestock farmer	PP patients
Test	Var	Mean (95 % CrI)	Mean (95 % CrI)
	Prev	1.1 (0.1–2.8)	1.7 (0.2–4.1)
iELISA	Se	68.1 (54.2–80.7)	69.6 (56.0–81.6)
	Sp	98.8 (97.7–99.5)	98.4 (97.0–99.3)
Rose Bengal	Se	79.4 (59.5–95.0)	79.2 (60.3–94.8)
	Sp	97.9 (96.1–99.3)	98.2 (96.4–99.5)
Standard tube agglutination	Se	80.5 (63.1–93.8)	80.6 (63.6–93.8)
	Sp	97.8 (96.2–98.9)	97.9 (96.4–99.2)

*PP* prolonged pyrexia, *Var* variable, *Prev* prevalence, *CrI* credibility interval, *Se* sensitivity, *Sp* specificity

### Positive and negative predictive values

The PPV and NPV of three serological tests were shown in Table 4. The PPV of three serological tests varied from 27.9 % (95 % CrI 3.6–62.0) to 36.3 % (95 % CrI 5.6–70.5) in livestock farmers and 39.8 % (95 % CrI 6.0–75.2) to 42.7 % (95 % CrI 6.4–83.2) in PP patients. The NPV of all three tests were very high and more than 99.5 %.

**Table 4:** The positive and negative predictive values of three serological tests

Test	Variable	Livestock farmer (95 % CrI) Prevalence: 1.1 %	PP patients (95 % CrI) Prevalence: 1.7 %
iELISA	PPV (%)	36.3 (5.6–70.5)	41.4 (6.6–76.1)
	NPV (%)	99.6 (98.9–99.9)	99.5 % (98.6–99.9)
Rose Bengal	PPV (%)	29.9 (3.6–69.5)	42.7 (6.4–83.2)
	NPV (%)	99.8 (99.2–99.9)	99.6 % (98.8–99.9)
Standard tube agglutination	PPV (%)	27.9 (3.6–62.0)	39.8 (6.0–75.2)
	NPV (%)	99.8 (99.3–99.9)	99.6 % (98.9–99.9)

*PPV* positive predictive value, *NPV* negative predictive value, *CrI* credibility interval

### Results of sensitivity analyses

The true prevalences and specificities of all three tests obtained from the different models of sensitivity analyses were similar. Whereas the estimated sensitivities varied in two models and yielded wider confidence intervals. But as their 95 % credibility intervals overlap, the observed differences were not statistically important (data not shown).

## Discussion

Data on the true prevalence of brucellosis, characteristics of three serological tests in livestock farmers, and PP patients from Bangladesh are provided.

A Bayesian latent class evaluation was used to estimate the true prevalence of brucellosis in livestock farmers, PP patients, and at the same time to evaluate three conditionally dependent serological tests. Bangladesh has to be considered to be endemic for brucellosis but with a very low prevalence in animals and humans [19]. In areas of low endemicity, the risk for human infection originates either from consumption of non-pasteurized dairy products or occupation threatening veterinarians, abattoir workers, farmers, and laboratory personnel. In this study, it was possible to estimate the true prevalence for livestock farmers. Sample sizes for other occupational groups were too small to do so, and the method of collection was also non-random. This is another limitation of this study. However, livestock farmers are a promising study group as almost 85 % of rural households own animals and 75 % of the population rely to some extent on livestock for their livelihood [20, 21]. The true prevalence for this group was estimated to be 1.1 %. Brucellosis is a pyrexic disease. As such, it was of interest to investigate also PP patients due to the assumption that brucellosis may be regularly ignored or misdiagnosed. If so, the number of pyrexic patients infected with brucellosis is considered to be valuable information not only for family physicians but also for policy makers. In this study, we focused on PP patients because these patients take antipyretic drugs and antibiotics inappropriate for brucellosis, and see doctors only if recovery does not occur. Among PP patients, 1.7 % were found to be positive for brucellosis which confirms our assumption that brucellosis is ignored or misdiagnosed by physicians in Bangladesh.

Both in livestock farmers and PP patients, the performance of all three serological tests was similar. RBT does not need sophisticated infrastructure or extensive training; it is amazingly cheap and fast. For the Bangladesh setting, RBT is the test of choice. For some endemic countries, authors reported specificity problems of the RBT [22, 23]. In order to overcome this specificity problem, Diaz et al. [24] recommended a modified protocol, i.e., predilution of serum >1:4. Interestingly, we found almost the same performance for the RBT as described by Diaz et al. [24] but without any modification. If the prevalence of a disease is very low as it is in Bangladesh, there will be lower positive and higher negative predictive values for the tests [25]. We have also observed lower positive predictive values of the serologic tests. The highest positive predictive value of RBT in PP patients was 42.7 % indicating that 42.7 % test positive patients truly have the disease and the remaining are falsely positive. The positive predictive value may be increased by applying a second test with high specificity and/or by testing patients having history of exposure with known risk factors like contact with animals, consumption of raw milk, and/or having some symptoms like pyrexia, arthralgia, backache, etc.

Anti-Brucella antibodies, especially IgG, can persist for a longer period of time, i.e., several months even after recovery from disease [26]. For that reason, the presence of anti-Brucella antibodies cannot reflect the true disease status as described above. Thus, diagnosis should be confirmed in a sero-positive patient by the presence of at least one of the clinical symptoms and signs suggestive of brucellosis like pyrexia, arthralgia, headache, backache, hepatomegally, splenomegally, etc. [23, 27]. Applying a more specific test genus or species-specific real time PCR may also be performed [28] to avoid unjustified costs, drug toxicity, and masking of other potentially dangerous diseases like tuberculosis, which are also endemic in Bangladesh.

For a quantitative test, the sensitivity or specificity depends largely on the cut-off value chosen and other factors like endemicity, status and duration of infection, persistence of antibody titres after treatment, presence of cross-reacting pathogens etc. [25]. The cut-off value of the iELISA (≥20U/ml) used in our study seems to be appropriate to avoid false positives as its specificity was very high ranging from 99.3 to 99.6 %. WHO and OIE provide guidelines for STAT and RBT standardization, but not for the iELISAs. So, the iELISA test kits provided by different companies are not standardized and it is difficult to compare the results of different studies due to different cutoffs used. In general, a “new” cutoff should be determined under local conditions to avoid false positives.

Like many other authors, we have considered a STAT titre of 1:160 as positive [17, 22]. As already mentioned earlier, in regions where brucellosis is endemic, a large proportion of the population may have persistent Brucella-specific antibody titres. In this scenario, some authors recommend to use STAT titres of 1:320 or higher to avoid false positives [28, 29]. However, in our study, a STAT titre of 1:160 seems to be appropriate as this titre resulted in specificity ranging from 98.2 to 98.8 % indicating good fit for our setting.

The Bayesian latent class evaluation of diagnostic tests requires an assessment of variations in the prior information on the estimated parameters using a sensitivity analysis [30]. Our sensitivity analysis indicated that the use of diffused priors had no relevant influence on the estimated prevalence and test sensitivities and specificities.

## Conclusions

Based on the performance of the three serological tests validated in a setting where the prevalence of brucellosis is low in humans and animals, no single test can be recommended for routine diagnosis of human brucellosis in Bangladesh. Applying a second test with high specificity and/or testing patients with the history of exposure with known risk factors and/or testing patients having some clinical signs and symptoms of brucellosis may increase the positive predictive value of the serologic tests.

## Additional file

Additional file 1: Appendix A. (DOCX 23 kb)

## Abbreviations

CrI: Credibility interval
DIC: Deviance Information Criterion
iELISA: Indirect ELISA
MMC: Mymensingh Medical College
NPV: Negative predictive value
PP: Prolonged pyrexia
PPV: Positive predictive value
RBT: Rose Bengal Test
sLPS: Anti-Brucella smooth lipopolysaccharide
STAT: Standard tube agglutination test
