# Supplementary material for: Latent class evaluation of three serological tests for the diagnosis of human brucellosis in Bangladesh
Source: Trop Med Health. 2016 Oct 4;44:32. doi: 10.1186/s41182-016-0031-8 (PMC5048465; doi:10.1186/s41182-016-0031-8)
Supplement: Additional file 1: — Appendix A. (DOCX 23 kb) [file 41182_2016_31_MOESM1_ESM.docx]

list(r1=c(2,0,0,0,3,1,3,326), n1=335, r2=c(6,0,0,0,1,0,1,292), n2=300)

model

{

r1[1:8] ~ dmulti(p1[1:8], n1)

r2[1:8] ~ dmulti(p2[1:8], n2)

## p1[1] & p2[1]= p(111); p1[8] & p2[8] = p(000); pr = true prevalence

p1[1] <- pr[1]*th1[2]*th1[4]*th1[8] + (1-pr[1])*(1-th1[3])*(1-th1[7])*(1-th1[15])

p1[2] <- pr[1]*th1[2]*th1[4]*(1-th1[8]) + (1-pr[1])*(1-th1[3])*(1-th1[7])*th1[15]

p1[3] <- pr[1]*th1[2]*(1-th1[4])*th1[9] + (1-pr[1])*(1-th1[3])*th1[7]*(1-th1[14])

p1[4] <- pr[1]*th1[2]*(1-th1[4])*(1-th1[9]) + (1-pr[1])*(1-th1[3])*th1[7]*th1[14]

p1[5] <- pr[1]*(1-th1[2])*th1[5]*th1[10] + (1-pr[1])*th1[3]*(1-th1[6])*(1-th1[13])

p1[6] <- pr[1]*(1-th1[2])*th1[5]*(1-th1[10]) + (1-pr[1])*th1[3]*(1-th1[6])*th1[13]

p1[7] <- pr[1]*(1-th1[2])*(1-th1[5])*th1[11] + (1-pr[1])*th1[3]*th1[6]*(1-th1[12])

p1[8] <- pr[1]*(1-th1[2])*(1-th1[5])*(1-th1[11]) + (1-pr[1])*th1[3]*th1[6]*th1[12]

p2[1] <- pr[2]*th2[2]*th2[4]*th2[8] + (1-pr[2])*(1-th2[3])*(1-th2[7])*(1-th2[15])

p2[2] <- pr[2]*th2[2]*th2[4]*(1-th2[8]) + (1-pr[2])*(1-th2[3])*(1-th2[7])*th2[15]

p2[3] <- pr[2]*th2[2]*(1-th2[4])*th2[9] + (1-pr[2])*(1-th2[3])*th2[7]*(1-th2[14])

p2[4] <- pr[2]*th2[2]*(1-th2[4])*(1-th2[9]) + (1-pr[2])*(1-th2[3])*th2[7]*th2[14]

p2[5] <- pr[2]*(1-th2[2])*th2[5]*th2[10] + (1-pr[2])*th2[3]*(1-th2[6])*(1-th2[13])

p2[6] <- pr[2]*(1-th2[2])*th2[5]*(1-th2[10]) + (1-pr[2])*th2[3]*(1-th2[6])*th2[13]

p2[7] <- pr[2]*(1-th2[2])*(1-th2[5])*th2[11] + (1-pr[2])*th2[3]*th2[6]*(1-th2[12])

p2[8] <- pr[2]*(1-th2[2])*(1-th2[5])*(1-th2[11]) + (1-pr[2])*th2[3]*th2[6]*th2[12]

pr[1] ~ dbeta(1.57,29.19)##Prevalence in livestock farmers

th1[2] ~ dbeta(32.53,14.51)##Sensitivity of the first test (iLEISA)

th1[3] ~ dbeta(294.08,6.98)##Specificity of the first test (iELISA)

th1[4] ~ dbeta(143.50,10.09)## Probability to have a positive result for the second test (RBT) if the individual is seropositive and positive for the first test

th1[5] ~ dbeta(1,1)##Probability to have a positive result for the RBT if the individual is seropositive and negative for the iELISA

th1[6] ~ dbeta (1,1)##Probability to have a negative result for the RBT if the individual is seronegative and negative for the iLEISA

th1[7] ~ dbeta (1,1)##Probability to have a negative result for the RBT if the individual is seronegative and positive for the iELISA

th1[8] ~ dbeta (313.97,10.68)##Probability to have a positive result for the third test (STAT) if the individual is seropositive and positive for the iELISA and RBT

th1[9] ~ dbeta (1,1) ##Probability to have a positive result for the STAT if the individual is seropositive and positive for the iELISA and negative for the RBT

th1[10] ~ dbeta (1,1)##Probability to have a positive result for the STAT if the individual is seropositive and negative for the iELISA and positive for the RBT

th1[11] ~ dbeta (1,1)##Probability to have a positive result for the STAT if the individual is seropositive and negative for the iELISA and RBT

th1[12] ~ dbeta (999.99,6.02)##Probability to have a negative result for the STAT if the individual is seronegative and negative for the iELISA and RBT

th1[13] ~ dbeta (1,1)##Probability to have a negative result for the STAT if the individual is seronegative and negative for the iELISA and positive for the RBT

th1[14] ~ dbeta (1,1) ##Probability to have a negative result for the STAT if the individual is seronegative and positive for the iELISA and negative for the RBT

th1[15] ~ dbeta (1,1) ##Probability to have a negative result for the STAT if the individual is seronegative and positive for the iELISA and RBT

pr[2] ~ dbeta (1.57,29.19)

th2[2] ~ dbeta (32.53,14.51)

th2[3] ~ dbeta (294.08,6.98)

th2[4] ~ dbeta (143.50,10.09)

th2[5] ~ dbeta (1,1)

th2[6] ~ dbeta (1,1)

th2[7] ~ dbeta (1,1)

th2[8] ~ dbeta (313.97,10.683)

th2[9] ~ dbeta (1,1)

th2[10] ~ dbeta (1,1)

th2[11] ~ dbeta (`,1)

th2[12] ~ dbeta (999.99,6.02)

th2[13] ~ dbeta (1,1)

th2[14] ~ dbeta (1,1)

th2[15] ~ dbeta (1,1)

se1[1] <- th1[2]

sp1[1] <- th1[3]

se1[2] <- th1[2]*th1[4] + (1-th1[2])*th1[5]

sp1[2] <- th1[3]*th1[6] + (1-th1[3])*th1[7]

se1[3] <- th1[2]*(th1[4]*th1[8]+(1-th1[4])*th1[9]) + (1-th1[2])*(th1[5]*th1[10]+(1-th1[5])*th1[11])

sp1[3] <- th1[3]*(th1[6]*th1[12]+(1-th1[6])*th1[13]) + (1-th1[3])*(th1[7]*th1[14]+(1-th1[7])*th1[15])

se2[1] <- th2[2]

sp2[1] <- th2[3]

se2[2] <- th2[2]*th2[4] + (1-th2[2])*th2[5]

sp2[2] <- th2[3]*th2[6] + (1-th2[3])*th2[7]

se2[3] <- th2[2]*(th2[4]*th2[8]+(1-th2[4])*th2[9]) + (1-th2[2])*(th2[5]*th2[10]+(1-th2[5])*th2[11])

sp2[3] <- th2[3]*(th2[6]*th2[12]+(1-th2[6])*th2[13]) + (1-th2[3])*(th2[7]*th2[14]+(1-th2[7])*th2[15])

#ppv ELISA in livestock farmer

ppveliv<- (se1[1]*pr[1])/( se1[1]*pr[1]+(1-sp1[1])*(1-pr[1]))

#ppv ELISA in pyrexic patient

ppvelpy<-(se2[1]*pr[2])/ <-(se2[1]*pr[2]+(1-sp2[1])*(1-pr[2]))

#ppv RBT in livestock farmer

ppvrbli<-(se1[2]*pr[1])/ (se1[2]*pr[1]+(1-sp1[2])*(1-pr[1]))

#ppv RBT in pyrexic patient

ppvrbpy<-( se2[2]*pr[2])/( se2[2]*pr[2]+(1-sp2[2])*(1-pr[2]))

#ppv of STAT in livestock farmer

ppvstli<-(se1[3]*pr[1])/( se1[3]*pr[1]+(1-sp1[3])*(1-pr[1]))

#ppv STAT in pyrexic patient

ppvstpy<- (se2[3]*pr[2])/( se2[3]*pr[2]+(1-sp2[3])*(1-pr[2]))

#NPV ELISA in livestock farmer

npveliv<- (sp1[1]*(1-pr[1]))/( (1-se1[1])*pr[1]+sp1[1]*(1-pr[1]))

#NPV ELISA pyrexoc patient

npvelpy<- (sp2[1]*(1-pr[2]))/((1-se2[1])*pr[2]+sp2[1]*(1-pr[2]))

#NPV RBT livestock farmer

npvrbliv<-(sp1[2]*(1-pr[1]))/ (1-se1[2])*pr[1]+sp1[2]*(1-pr[1]))

#NPV RBT pyrexic patient

npvrbpy<-( sp2[2]*(1-pr[2])) /((1-se2[2])*pr[2]+sp2[2]*(1-pr[2]))

#NPV STAT in livestock farmer

npvstliv<-(sp1[3]*(1-pr[1]))/ ((1-se1[3])*pr[1]+sp1[3]*(1-pr[1]))

#NPV of STAT in pyrexic patient

npvstpy<-(sp2[3]*(1-pr[2]))/ ((1-se2[3])*pr[2]+sp2[3]*(1-pr[2]))

for ( i in 1:8)

{

d1[i] <- r1[i]*log(max(r1[i],1)/(p1[i]*n1))

}

G0 <- 2*sum(d1[])

r11[1:8] ~ dmulti(p1[1:8],n1)

for (i in 1:8)

{

d11[i] <- r11[i]*log(max(r11[i],1)/(p1[i]*n1))

}

Gt <- 2*sum(d11[])

bayesp1 <- step(G0 - Gt)

for ( i in 1:8)

{

d2[i] <- r2[i]*log(max(r2[i],1)/(p2[i]*n2))

}

G10 <- 2*sum(d2[])

r22[1:8] ~ dmulti(p2[1:8],n2)

for (i in 1:8)

{

d22[i] <- r22[i]*log(max(r22[i],1)/(p2[i]*n2))

}

G1t <- 2*sum(d22[])

bayesp2 <- step(G10 - G1t)

for ( i in 1:3)

{

sediff[i]<-se1[i]-se2[i]

spdiff[i]<-sp1[i]-sp2[i]

}

prevdiff<-pr[1]-pr[2]

for ( i in 1:1)

{

sediff1elirb[i]<-se1[1]-se1[2]

sediff1elisat[i]<-se1[1]-se1[3]

sediff1rbsat[i]<-se1[2]-se1[3]

sediff2elirb[i]<-se1[1]-se1[2]

sediff2elisat[i]<-se1[1]-se1[3]

sediff2rbsat[i]<-se1[2]-se1[3]

spdiff1elirb[i]<-sp1[1]-sp1[2]

spdiff1elisat[i]<-sp1[1]-sp1[3]

spdiff1rbsat[i]<-sp1[2]-sp1[3]

spdiff2elirb[i]<-sp1[1]-sp1[2]

spdiff2elisat[i]<-sp1[1]-sp1[3]

spdiff2rbsat[i]<-sp1[2]-sp1[3]

}

}
